# Supplementary material for: Dimethylglycine Can Enhance the Cryopreservation of Red Blood Cells by Reducing Ice Formation and Oxidative Damage
Source: Int J Mol Sci. 2023 Apr 3;24(7):6696. doi: 10.3390/ijms24076696 (PMC10095525; doi:10.3390/ijms24076696)
Supplement: Supplementary file 1 [file ijms-24-06696-s001.zip › ijms-2243305-supplementary.pdf]

International Journal of *Molecular Sciences*

Article

Dimethylglycine Can Enhance the Cryopreservation of Red Blood Cells by Reducing Ice Formation and Oxidative Damage

Yuying Hu [1,†], Xiangjian Liu [1,†], Marlene Davis Ekpo [1], Jiangming Chen [1], Xiaoxiao Chen [1], Wenqian Zhang [1], Rui Zhao [1], Jingxian Xie [1], Yongju He [2] and Songwen Tan [1,*]

[1] Xiangya School of Pharmaceutical Sciences, Central South University, Changsha 410013, China
[2] School of Materials Science and Engineering, Central South University, Changsha 410013, China

Experimental Section

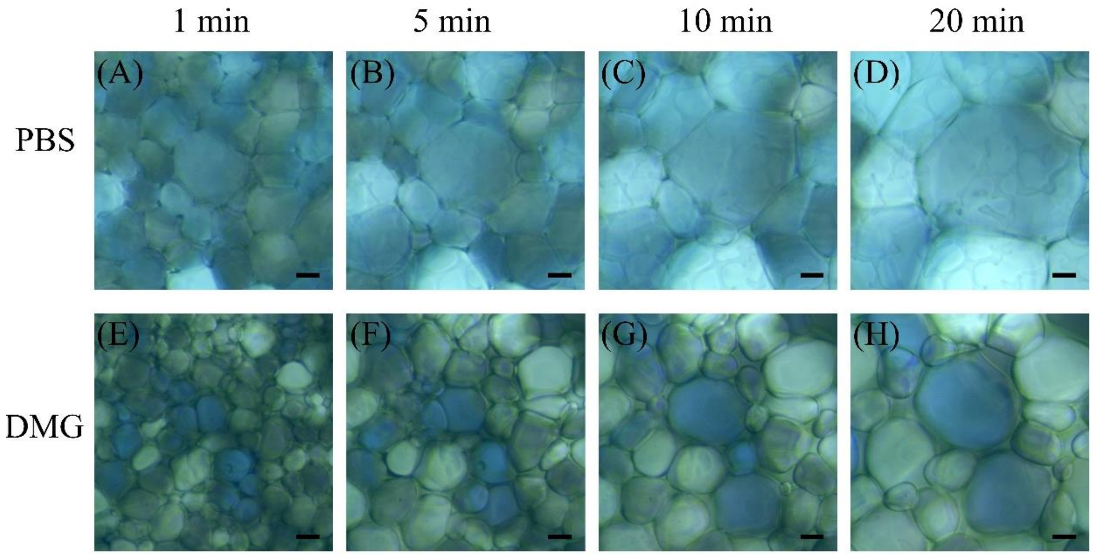

Figure S1. Ice recrystallization inhibition assays. Additional cryomicroscopic images of PBS at different time highlighting ice crystal growth were shown in (A) 1 min, (B) 5 min, (C) 10 min, and (D) 20 min. The representative images of ice crystals of DMG at different time were shown in (E) 1 min, (F) 5 min, (G) 10 min, and (H) 20 min. The concentration of DMG was 4%. Scale bar = 10 μm.

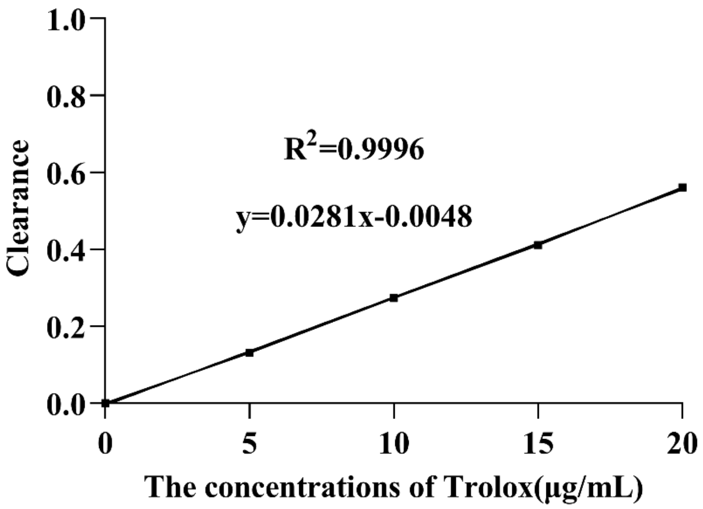

Figure S2. The standard curve of clearance and concentrations of Trolox.
